# Current Pathways of Care Continue to Fail Those With a Diagnosis of Endometriosis

**DOI:** 10.1111/1471-0528.18245

**Published:** 2025-06-16

**Authors:** Katy Vincent, Andrew W. Horne

**Affiliations:** ^1^ Nuffield Department of Women's and Reproductive Health, University of Oxford; ^2^ Centre for Reproductive Health Institute of Regeneration and Repair, University of Edinburgh Edinburgh UK

**Keywords:** endometriosis: basic science, endometriosis: drug treatement, endometriosis: surgery, pelvic pain

Endometriosis is a chronic, oestrogen‐dependent, inflammatory condition that is associated with disabling pelvic pain and infertility [1]. It affects ~10% of those born female, and recent data show that within the NHS in England alone, almost 2500 women per month receive the diagnosis [2]. Current UK clinical practice for the management of endometriosis‐associated pain aligns with the recommendations of both NICE [3] and ESHRE [4], focusing on simple analgesia, hormonal therapies and surgical removal (excision or ablation) of lesions. There has been increasing awareness of endometriosis among both primary and secondary care clinicians, researchers and the public over recent years, including high‐profile media coverage. Much of the dialogue focuses on diagnostic delay, which remains between 5–12 years from onset of symptoms throughout the developed world [5]. These narratives frequently highlight the ongoing dismissal of women's pain and wider‐reaching medical misogyny as key contributors. However, there have been two recent largescale UK reports which paint a stark picture of the challenges which also face those who ***have*** received a diagnosis and call into question our approach to the subsequent management of the condition.

## 1 2024 NCEPOD Report

In July 2024, the National Confidential Enquiry into Patient Outcome and Death (NCEPOD) published their review of the quality of healthcare provided to adults diagnosed with endometriosis [6]. This enquiry combined data from multiple sources (623 clinician questionnaires, 167 organisational questionnaires, review of 309 sets of case notes, 941 responses to a patient survey and 137 responses to a clinician survey) to assess the pathway and quality of care provided by the NHS in England, Wales and Northern Ireland between 1 February 2018 and 31 July 2020 to those aged 18 and over with an endometriosis diagnosis. Aligned with NICE/ESHRE guidance, a large proportion (77.9%) of patients had been prescribed hormonal treatment in primary and/or secondary care. Appropriately, many of those who were not offered hormonal therapy were trying to conceive. However, 42.8% reported no improvement with hormonal medication. To be included in this study, patients had to have had a laparoscopy during their index admission and thus we cannot use these data to determine any information about rates of surgery in the UK. However, given the central role of surgery in the current management of endometriosis, one of the most striking findings of this report was that only 17.3% of patients were satisfied with the results of their primary surgery. It is also surprising, given that pain was the primary presenting symptom for the majority (77.7%), that only just over half (54.4%) of patients were prescribed pain medication. This may of course represent an assumption that simple analgesics could be bought over the counter. Despite good evidence that chronic pain (including in those with endometriosis) is not well managed by opiates and the known challenges of long‐term opioid use, almost a third of patients (31.6%) were prescribed opioids. Moreover, 8.7% were prescribed gabapentinoids, Class C controlled drugs for which there is no evidence supporting benefit in endometriosis‐associated pain. Given the high rates of dissatisfaction with surgical treatment, persistent symptoms with hormonal treatment, and use of pain medication with significant potential for addiction and abuse, it is disappointing that only 7.4% saw a pain medicine specialist.

## 2 2025 ONS Bulletin

It is well established that the financial burden of endometriosis is not just to the health care system but also to the individual themselves. Given that receiving a diagnosis should be the gateway to the implementation of optimum treatment and thus a reduction in the impact of the disease, the report published in February 2025 by the English Office for National Statistics (ONS) [7] is particularly concerning. They explored the impact of a (surgical) diagnosis on both monthly pay and employee status, comparing the 2 years prior to diagnosis with the following 5 years after diagnosis. It is perhaps not surprising that monthly pay dropped in the first 3 months post‐diagnosis, likely reflecting unpaid sick leave and/or a reduction in choosing to work any/additional hours for those on zero hour/standard contracts respectively, as individuals recovered from their surgery. Reassuringly, earnings then returned to pre‐diagnostic levels from 4–12 months post‐surgery. What is more concerning is that beyond 12 months both monthly pay and the probability of being in paid employment continued to decrease throughout the analysis period (up to 5 years post‐surgery). The analysis considered factors known to impact work and earning potential, such as age, changes in the labour market and births. Thus, combined with the fact that pain rather than fertility is the predominant indication for surgery, it is unlikely that these findings are completely explained by pregnancies after endometriosis treatment. Qualitative work is needed to understand how receiving a diagnosis of endometriosis may change an individual's perception of themselves as capable of higher earning potential or even of working at all. Furthermore, despite efforts to improve understanding of endometriosis in the workplace and how employees can be better supported, such as the Endometriosis UK Endometriosis Friendly Employer Scheme (https://www.endometriosis‐uk.org/endometriosis‐friendly‐employer‐scheme), we continue to hear from our patients about challenges they encounter at work. The role of employer perceptions thus also needs to be explored.

## 3 Is It Time for an Alternative Approach to Endometriosis‐Associated Pain?

Taken together, these two reports highlight that once a diagnosis is made, current management approaches leave many women with endometriosis with persisting symptoms, which continue to impact on their life. Whilst the NCEPOD report does highlight organisational factors that could improve patient care and employers may have a role in improving earning potential, we believe that this data further supports the urgent need to revisit our strategies and recommendations for the management of endometriosis‐associated pain.

The traditional view of endometriosis has focused on the roles of lesion‐related factors, such as inflammation and fibrosis as drivers of pain. With this in mind, strategies to suppress or remove the lesions make sense. However, we know that chronic pain is complex. For some people, ongoing activation of peripheral nociceptors continues to drive pain and this would be considered as ‘nociceptive’ pain. However, for others, alterations in the function of the peripheral and/or central nervous system can generate or amplify pain even without the need for ongoing peripheral nociceptive input (‘neuropathic’ or ‘nociplastic’ pain). In the context of endometriosis, the combination of inflammation, neoinnervation and surgical procedures may all potentially contribute to the development of a neuropathic‐like component to pain. Mechanisms by which nociplastic pain is generated are less well understood but relevant processes include systemic inflammation, repeated episodes of pain (including dysmenorrhoea) and alterations in stress response systems. Studies suggest that up to 40% of those with endometriosis‐associated pain have a neuropathic‐like component and a similar proportion have features consistent with a nociplastic mechanism (e.g., widespread pain, fatigue and ‘brain fog’) [8]. The presence of these non‐nociceptive mechanisms may explain, at least in part, the poor response to treatments targeting the lesions themselves.

If we accept that our current solely nociceptive approach to endometriosis pain management is inappropriate, there are two key questions that need addressing to determine the correct approach:

1. How do we best manage non‐nociceptive pain in association with endometriosis?
2. How do we identify those who may still benefit from a lesion‐focused approach?

Recommended treatments for neuropathic pain are medical: the use of anti‐depressants (e.g., tricyclics and SNRIs), gabapentinoids and other anti‐epileptics (e.g., lamotrigine), although all of these have relatively large numbers needed to treat (NNT) to see benefit [9]. To date, none of these drugs have been trialled in endometriosis‐associated pain [8]. It is clear from the NCEPOD report that antidepressants and gabapentinoids are used for these women (> 20% had been prescribed antidepressants). However, given how few had seen a pain medicine specialist, it is unclear why these medications were selected. Psychological comorbidities are very common in those with endometriosis‐associated pain, and thus these drugs may have been suggested for this reason rather than because the history or examination findings suggested a neuropathic component. They can also be considered for nociplastic pain but the optimal approach to the management of this type of chronic pain is usually multimodal, combining medication with pain psychology, physiotherapy and lifestyle modifications [9]. Clinical trials of some components of these approaches have been undertaken in endometriosis‐associated pain but often in relatively small cohorts or without adequate control arms. There is very limited literature exploring the benefit of a multidisciplinary pain management approach despite this being central to the care of many other types of chronic pain [8].

Whilst we acknowledge that diagnosing neuropathic pain can be challenging, it is possible to make a good assessment of the likely type of chronic pain present from a detailed history and (pain‐focused) examination. Validated questionnaires are also available that assess for a likely neuropathic or nociplastic component, but these were not developed for those with endometriosis. Furthermore, it is acknowledged that more than one form of chronic pain can coexist. For example, just because a patient has features consistent with a nociplastic component, this does not exclude the possibility of a nociceptive component being present too. The challenge is therefore to understand whether there is still benefit in treating the nociceptive component. The balance of benefit to risk is likely to be different when thinking about hormonal versus surgical therapies to target the lesions. Many patients with endometriosis‐associated pain will have other unpleasant or life‐impacting cyclical symptoms (e.g., heavy menstrual bleeding) and/or require contraception. Thus, a hormonal medication (if acceptable and tolerated) may be a sensible adjunct to any other approaches put in place. Surgery, however, is associated with considerably greater risk and there is increasing evidence in both pelvic and other forms of chronic pain that those with nociplastic pain are less likely to see benefit from surgery [8]. Importantly, surgery is itself associated with a risk of new chronic pain (post‐surgical pain), and this risk appears to be present for both laparoscopic and open procedures. Counselling about the risk of new post‐surgical pain is essential.

**FIGURE 1:**
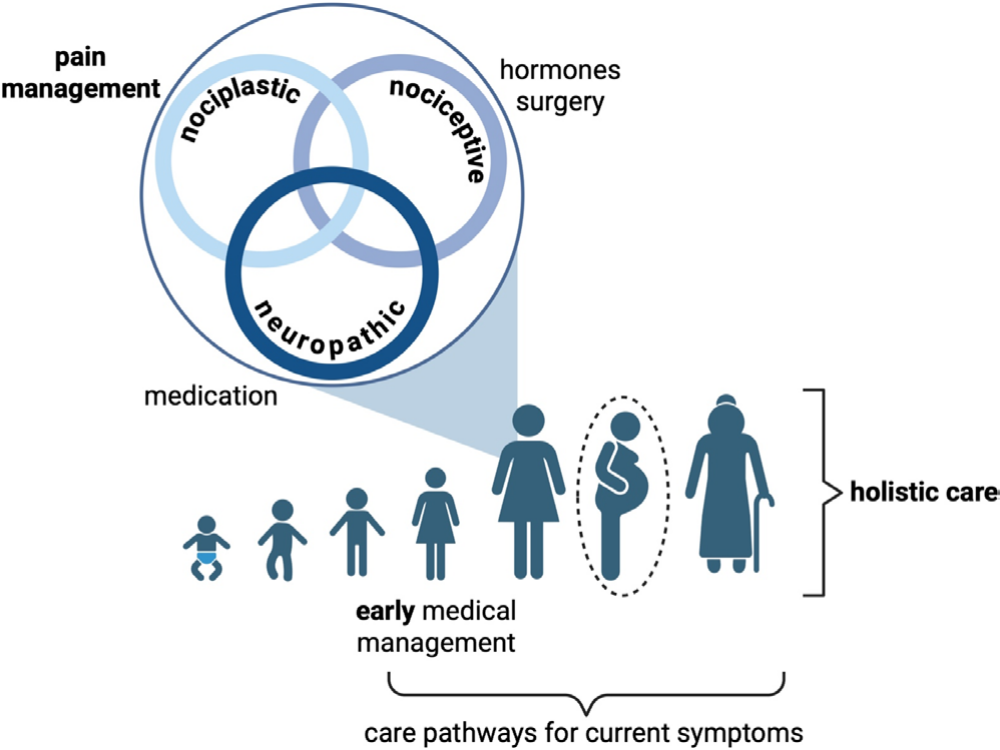
An alternative approach to the management of endometriosis‐associated pain across the life course. Pregnancy is within a dashed circle as this may not be part of an individual's life course through choice or otherwise.

So, what would a care pathway look like that focussed on the pain instead of primarily considering the lesions? (Figure 1) We believe that following a number of recommendations in the NCEPOD report [6] would be a good start:

- Initiate medical management early, with a low threshold for prescribing both hormonal and analgesic therapies.
- Refer to services that specialise in pain management (both pharmacologically and otherwise)
- Ensure that holistic care is provided, managing all symptoms (both physical and psychological) that impact on quality of life.
- Recognise that endometriosis is a chronic condition and ensure that care pathways take into consideration the recurrent nature of symptoms.

There are also clearly areas where further research is needed, not least to identify strategies to better stratify patients allowing the identification of the appropriate therapeutic options. However, there is already enough evidence that endometriosis‐associated pain is similar in so many ways to other forms of chronic pain [8, 10] that we should feel confident in taking a pain‐focussed approach. In fact, the 2021 UK NICE guidance for chronic pain specifically highlights that its recommendations may also apply to endometriosis‐associated pain [11]. However, for such a pathway to be successful would require clinicians to be fully aware of the limitations (and harms) of surgical treatment to endometriosis and for the rhetoric within the media and social media to move away from a focus on diagnostic delay as the major challenge and ‘excision surgery’ as the optimum solution.

## Author Contributions

K.V. conception and writing up the manuscript. A.W.H. conception and writing up the manuscript.

## Disclosure

K.V. reports payments to her institution for consultancy and talks from Bayer Healthcare, Gedeon Richter, Reckitt and Gesynta.

A.W.H.'s institution has received honoraria for consultancy from Roche Diagnostics, Gesynta and Joii, and he has received lecture fees from Theramex and Gedeon Richter. A.W.H.'s institution has received grant funding from Roche Diagnostics. He is a trustee of Endometriosis UK and president‐elect of the World Endometriosis Society.

## Conflicts of Interest

K.V. declares research funding from UKRI, NIHR, NIH US, and honoraria for consultancy and talks and associated travel expenses paid to her institution from Gedeon Richter, Gesynta, Reckitts and Eli Lilly. A.W.H. declares grant funding from UKRI, NIHR, CSO, Wellbeing of Women, Roche Diagnostics and payment to his institution for consultancy from Roche Diagnostics, Gesynta and Joii. A.W.H. has received payment for a lecture from Theramex and Gedeon Richter.

## Data Availability

Data sharing is not applicable to this article as no new data were created or analyzed in this study.
